# Treatment of patients with metastatic epidural spinal cord compression using an enhanced recovery after surgery program

**DOI:** 10.3389/fcell.2023.1183913

**Published:** 2023-05-12

**Authors:** Mingxing Lei, Wenjing Zheng, Yuncen Cao, Xuyong Cao, Xiaolin Shi, Xiuyun Su, Yaosheng Liu

**Affiliations:** ^1^ Senior Department of Orthopedic Surgery, The Fourth Medical Center of PLA General Hospital, Beijing, China; ^2^ Chinese PLA Medical School, Beijing, China; ^3^ Hainan Hospital of PLA General Hospital, Beijing, China; ^4^ Department of Chemical Poisoning Treatment, The Fifth Medical Center of PLA General Hospital, Beijing, China; ^5^ Department of Hematology, The Fifth Medical Center of PLA General Hospital, Beijing, China; ^6^ Department of Orthopedic Surgery, The Fifth Medical Center of PLA General Hospital, Beijing, China; ^7^ Department of Orthopedic Surgery, The Second Affiliated Hospital of Zhejiang Chinese Medical University, Hangzhou, China; ^8^ Intelligent Medical Innovation institute, Southern University of Science and Technology Hospital, Shenzhen, China; ^9^ National Clinical Research Center for Orthopedics, Sports Medicine & Rehabilitation, PLA General Hospital, Beijing, China

**Keywords:** metastatic epidural spinal cord compression, enhanced recovery after surgery, surgical outcome, mental health, spine metastases

## Abstract

**Purpose:** The aims of this study were to introduce a new medical, pathway based on the concept of “enhanced recovery after surgery” (ERAS) for patients with metastatic epidural spinal cord compression (MESCC), and to test whether the ERAS program could improve clinical metrics among such patients.

**Methods:** Data from patients with MESCC (*n* = 98), collected between December 2016 and December 2019 (Non-ERAS cohort), and from 86 patients with metastatic epidural spinal cord compression collected between January 2020 and December 2022 (ERAS cohort), were retrospectively analyzed. Patients were treated by decompressive surgery combined with transpedicular screw implantation and internal fixation. Patient baseline clinical characteristics were collected and compared between the two cohorts. Surgical outcomes analyzed included operation time; intraoperative blood loss; postoperative length of hospital stay; time to ambulation, regular diet, urinary catheter removal, and radiation therapy; perioperative complications; anxiety; depression; and satisfaction with treatment.

**Results:** No significant differences in clinical characteristics were found between the non-ERAS and enhanced recovery after surgery cohorts (all *p* > 0.050), indicating that the two cohorts were comparable. Regarding surgical outcomes, the enhanced recovery after surgery cohort had significantly less intraoperative blood loss (*p* < 0.001); shorter length of postoperative hospital stay (*p* < 0.001); shorter time to ambulation (*p* < 0.001), regular diet (*p* < 0.001), urinary catheter removal (*p* < 0.001), radiation administration (*p* < 0.001), and systemic internal therapy (*p* < 0.001); lower perioperative complication rate (*p* = 0.024); less postoperative anxiety (*p* = 0.041); and higher score for satisfaction with treatment (*p* < 0.001); whereas operation time (*p* = 0.524) and postoperative depression (*p* = 0.415) were similar between the two cohorts. Compliance analysis demonstrated that ERAS interventions were successfully conducted in the vast majority of patients.

**Conclusion:** The enhanced recovery after surgery intervention is beneficial to patients with metastatic epidural spinal cord compression, according to data on intraoperative blood loss; length of hospital stay; time to ambulation, regular diet, urinary catheter removal, radiation exposure, and systemic internal therapy; perioperative complication; alleviation of anxiety; and improvement of satisfaction. However, clinical trials to investigate the effect of enhanced recovery after surgery are needed in the future.

## 1 Introduction

Metastatic epidural spinal cord compression (MESCC) involves secondary compression of the spinal cord because of cancer metastasis to the spine or epidural space (Cole and Patchell, 2008), and is a significant source of morbidity among patients with cancer (Cole and Patchell, 2008). According to estimates, MESCC occurs among 5%–10% of patients with cancer (Cole and Patchell, 2008; L'Esperance et al., 2012). Patients with MESCC are characterized by symptoms including back pain and impaired sensory, motor, and even sphincter function (Cole and Patchell, 2008; Van den Brande et al., 2022). Rapid diagnosis and treatment are imperative, since MESCC is usually a medical emergency (Pipola et al., 2018). The treatment of MESCC requires multidisciplinary cooperation, and surgical decompression combined with postoperative radiotherapy, is considered the standard therapeutic intervention for selected patients (Vellayappan et al., 2018), as surgery plus adjuvant radiotherapy is clinically more effective than radiation alone (Fehlings et al., 2014).

Nevertheless, surgical treatment of patients with MESCC remains challenging for surgeons, as such patients experience a high rate of complications; recent reports indicate that 3%–35% of patients have complications (de Almeida Bastos et al., 2020; Echt et al., 2021). In addition, complications result in longer length of hospital stay, worse surgical outcomes, more readmissions, poorer quality of life, and a heavier economic burden (Bakar et al., 2016; Yahanda et al., 2019; Elsamadicy et al., 2021). The prevalence of need for surgery for spine metastases is rising, along with in-hospital complication rates (Yoshihara and Yoneoka, 2014). Up to 20% of patients with MESCC were found to have local recurrence after surgery (Echt et al., 2021). Open surgery involves a significant high volume of intra-operative blood loss, which is estimated at around 783 mL (Echt et al., 2021). Furthermore, open surgery can delay patients receiving radiation therapy (Echt et al., 2021). Therefore, it is imperative for surgeons to find new medical pathways to improve surgical outcomes among patients with MESCC.

Notably, enhanced recovery after surgery (ERAS) is an emerging concept from evidence-based medicine, which aims to improve patient surgical outcomes, shorten the length of hospital stay, reduce hospital costs, and optimize patient satisfaction with treatments (Wainwright et al., 2016). To date, ERAS has been introduced for patients undergoing spinal surgery, including lumbar spinal fusion (Debono et al., 2021), adolescent idiopathic scoliosis (Koucheki et al., 2021), lumbar minimally invasive transforaminal lumbar interbody fusion surgery (Feng et al., 2019), and intraspinal tumor surgery (Liu et al., 2020), with satisfactory effects. When applied in spinal procedures, the ERAS pathway is beneficial in reducing the duration of hospitalization, accelerating functional recovery, alleviating postoperative pain, reducing complications, decreasing opioid use, and relieving financial burden (Dietz et al., 2019; Elsarrag et al., 2019); however, the limited number of studies published, which vary significantly in terms of patient populations and ERAS protocol implementation, are obstacles to evaluation of the effectiveness of ERAS in spinal surgery (Dietz et al., 2019). In addition, use of ERAS interventions for patients with MESCC is rare.

The primary aim of this study was to introduce a new medical pathway, based on the concept of ERAS, for patients with MESCC. A series of surgery-related metrics were evaluated in the study, including: operation time; intraoperative blood loss; postoperative length of hospital stay; time to ambulation, regular diet, urinary catheter removal, radiation administration, and systemic internal therapy administration; perioperative complications; mental health (anxiety and depression); and satisfaction with treatment. The hypothesis tested in this study was that implementation of an ERAS program could promote faster recovery from surgery among patients with MESCC.

## 2 Patients and methods

### 2.1 Patients

**FIGURE 1:**
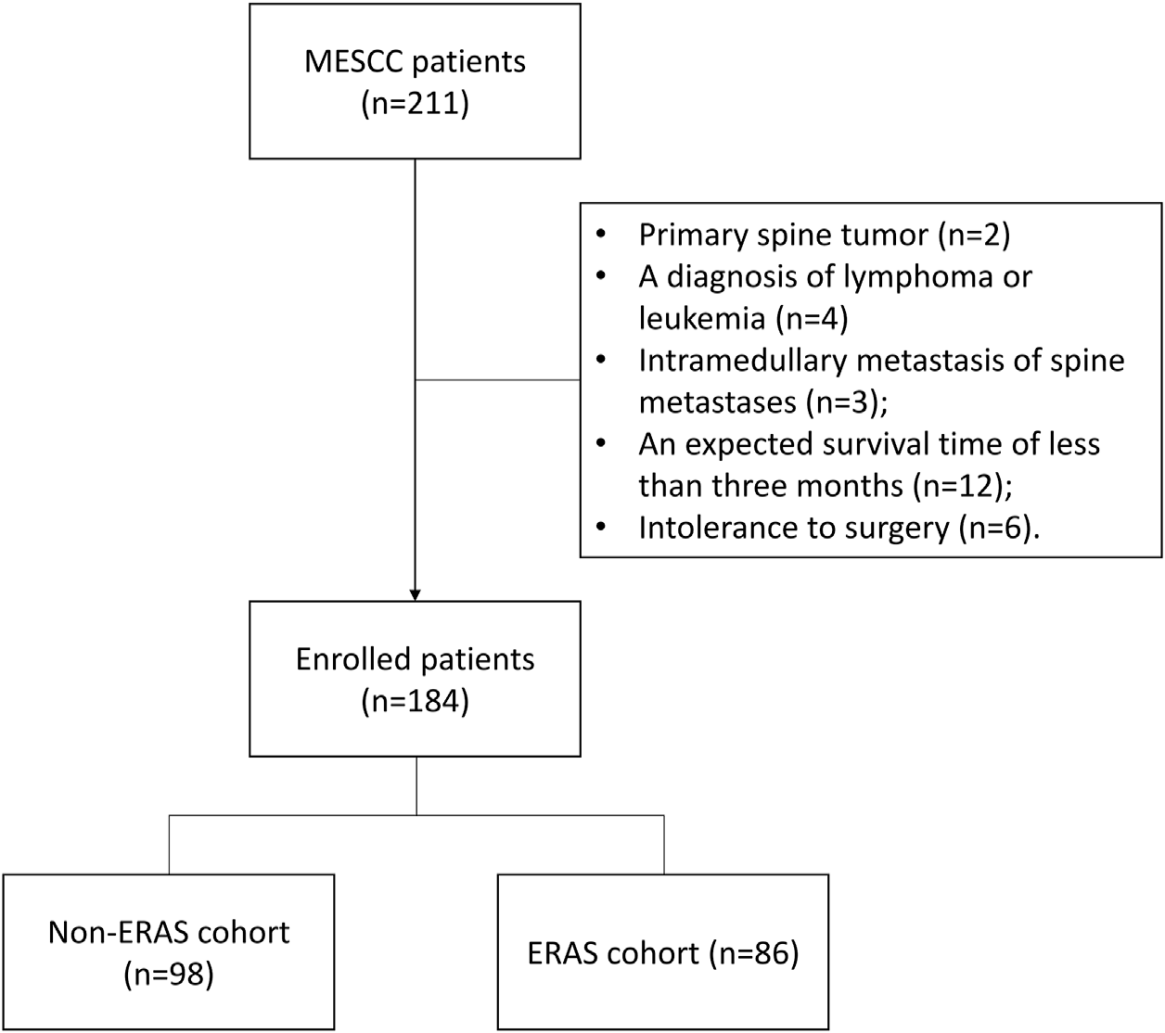
Flow diagram outlining patient enrollment.

A total of 184 patients with MESCC, treated with surgery at the orthopedic department of the Fifth Medical Center of PLA General Hospital (Beijing) between December 2016 and December 2022, were enrolled in this retrospective study. ERAS interventions were carried out for patients with MESCC from January 2020; therefore, 98 patients with MESCC treated between December 2016 and December 2019 were regarded as the non-ERAS cohort, and 86 treated between January 2020 and December 2022 served as the ERAS cohort in the study. Patients were classified into two groups, according to the presence of ERAS interventions. Patients were included for analysis, if they: had radiographical or histological evidence of MESCC; had one or more of the following symptoms: a) progressive local mechanical or radiating pain, b) progressive impairment of sensory function, c) impairment of lower limb motor function, or d) impairment of sphincter function; had an expected survival interval of more than 3 months; and were treated by circumferential decompression of the vertebral canal and partial intralesional excision of metastatic spine tumor using a posterolateral approach. Patients were excluded from the analysis if they met the following criteria: primary spine tumor; MESCC due to lymphoma or leukemia; intramedullary metastasis of spinal metastasis; previous surgery or radiation therapy for spinal metastases; and intolerant of surgery due to severe cardio-cerebrovascular disease, respiratory system disease, infection, liver or kidney insufficiency, or hemorrhagic and coagulative dysfunction. Figure 1 shows a flow diagram outlining patient enrollment. The Ethics Committee Board of PLA General Hospital approved the study and waived informed consent from patients, since all data analyzed were retrospective. The study was conducted in accordance with the Declaration of Helsinki.

### 2.2 ERAS program

**FIGURE 2:**
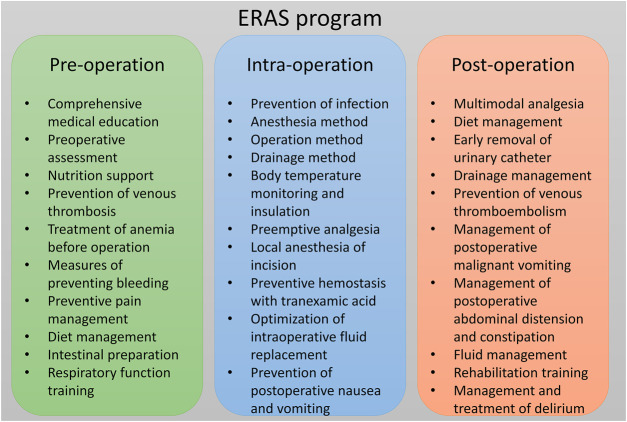
The main components of the ERAS program.

**FIGURE 3:**
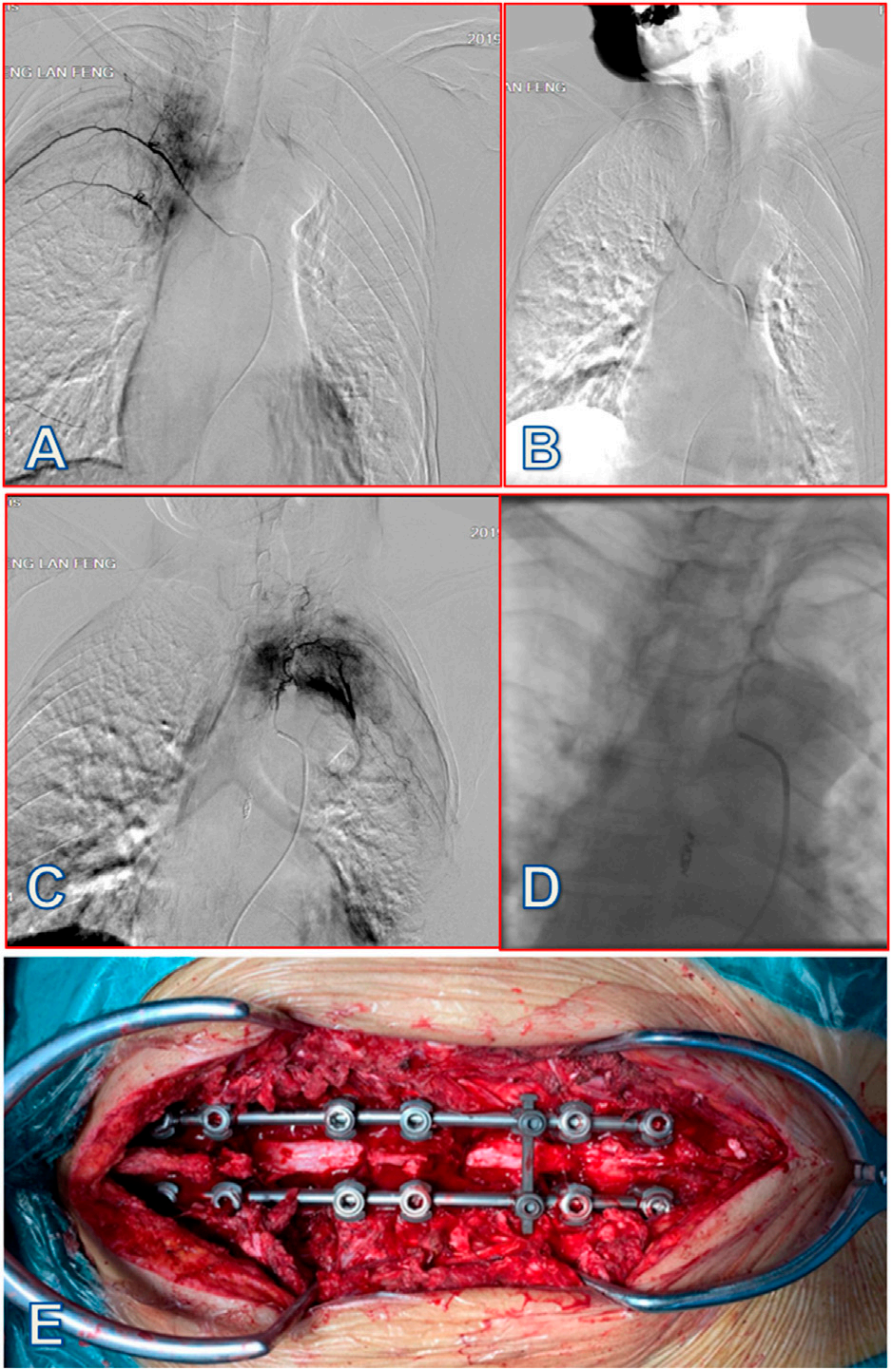
A 58-year-old female patient with lung cancer was diagnosed with MESCC due to T4 and T7 spine metastases, which resulted in incomplete paralysis. After preoperative arterial embolization, partial resection of the tumors was performed through a posterior approach, with circular decompression and internal fixation of the spinal canal. **(A)** After inserting a microcatheter into the right fifth intercostal artery tumor blood supply artery branch, under the guidance of a micro-guide wire, imaging showed that the diameter of the right fifth intercostal artery was thickened, and the distal branch and path were tortuous; tumor staining was visible. **(B)** After embolizing the right fifth intercostal artery tumor blood supply branch with gelatin sponge particles (20 mL), tumor staining disappeared. **(C)** After inserting a microcatheter into the left fifth intercostal artery tumor blood supply artery branch, under the guidance of the micro-guide wire, imaging showed that the diameter of the left fifth intercostal artery was thickened and the distal branch had a tortuous path; tumor staining was visible. **(D)** After embolizing the tumor supply branch of the left fifth intercostal artery with 20 mL gelatin sponge particles, tumor staining disappeared. **(E)** Partial resection of spine metastases at T4 and T7 via posterior approach from the thoracic spine with circular decompression and internal fixation of the vertebral canal, with little bleeding and clear visual field. Patient intraoperative blood loss was 300 mL, and time to ambulation was 24 h after surgery. At 5 days postoperative, patients received systemic internal therapy, and this patient had relatively favorable psychological health status and no surgery-related complications.

The ERAS program was established by multidisciplinary cooperation, with participants including spine surgeons, anesthesiologists, rehabilitation physicians, a radiotherapist, an oncologist, nutritionists, and nurses. Key perioperative metrics of interest and optimal practice guideline were identified by multidisciplinary discussion, supplemented by current spinal surgery ERAS reports. A new ERAS program was designed specifically for patients with MESCC after surgery (Figure 2). A case report is presented in Figure 3. The main contents of the ERAS program are described below.

#### 2.2.1 Preoperative items

##### 2.2.1.1 Comprehensive medical education

1) Patients were informed about the plans, risks, and complications of surgery, and introduced to measures to accelerate surgery recovery, as well as recommended discharge standards and follow-up guidelines during the perioperative period. 2) Health education manuals were distributed to patients, who were also advised to read the department’s education materials and watch the department’s ERAS education video and perioperative surgery rehabilitation exercise video. 3) Psychological assessment and counseling were conducted. In consultation with the psychosomatic medicine department, oral short-acting sedative and anti-anxiety drugs were recommended, if necessary. 4) A family WeChat group was established, to provide professional consultation at any time.

##### 2.2.1.2 Preoperative assessment

1) Anesthesia risk was evaluated using the American Association of Anesthesiologists guidelines.
2) Clinical characteristics, including Eastern Cooperative Oncology Group (ECOG) performance score, visual analogue scale pain score, anxiety and depression hospital anxiety and depression scale (HADS) score, nutritional status score, Caprini risk assessment scale for venous thrombosis, American Spinal Injury Association classification of spinal cord injury, Bilsky six-point classification of epidural spinal cord compression, and spinal instability tumor score, were evaluated.

##### 2.2.1.3 Nutrition support

1) Where preoperative albumin level was >35 g/L, oral or enteral nutrition support treatment was preferred, with emphasis on protein supplementation. 2) Patients with low nutritional risk were advised to eat high-protein and sugar-containing foods before surgery. Patients with high nutritional risk and cancer, were advised to add protein powder and immune-supporting nutrients, such as glutamine, arginine, and nucleotides, to the standard nutritional formula, for nutritional support. 3) Patients with MESCC and anorexia with progressive weight loss, a combination of enteral nutrition and off-site high nutrition was considered during the perioperative period.

##### 2.2.1.4 Prevention of venous thrombosis

1) Active/passive limb movement, graded compression elastic socks, or ICP pump were implemented. 2) Short-acting anticoagulants, such as low-molecular-weight heparin, were used for preventive treatment; low-molecular-weight heparin was stopped within 12–24 h before operation.

##### 2.2.1.5 Treatment of anemia before operation

1) If the patient was diagnosed with small cell hypochromic anemia, erythropoietin subcutaneous injection was recommended. 2) Iron or intravenous drip, oral folic acid, and multivitamin supplement were recommended for patients with anemia symptoms. 3) Before elective surgery, the above treatments for anemia were recommended for 5–6 days, and after surgery patients continued to receive treatment for anemia.

##### 2.2.1.6 Measures to prevent bleeding

1) Anti-vascular endothelial growth factor-targeted drugs, such as bevacizumab, and anticoagulants, antiplatelet therapy, or the thrombolytic drug, aspirin, were stopped and procedures delayed until the half-life duration of the relevant drug(s). 2) If platelet count was <50 × 10^9^/L, glucocorticoid and immunoglobulin were first injected, and if that was ineffective, platelet count was maintained at 50 × 10^9^/L during the perioperative period by infusion of 1–2 bags of platelets, collected before and after the operation. 3) If the primary tumor was renal cancer or prostate cancer, or the spinal metastatic tumor was judged to hyper-vascular by preoperative puncture biopsy or enhanced magnetic resonance imaging, selective arterial embolization was performed within 24–72 h before resection of the spinal tumor, if necessary.

##### 2.2.1.7 Preventive pain management

Before going to bed, gabapentin and paracetamol were administered orally, along with super-strong multimodal preventive analgesia.

##### 2.2.1.8 Diet management

1) Patients without gastrointestinal motility disorders were permitted to eat starchy foods, such as porridge and steamed bread 6 h before operation and take 400 mL a 12.5% of carbohydrate drink or intravenous infusion of 200 g glucose 2 h before operation. 2) For patients with diabetes, it was generally considered inappropriate to take nutrient solution 2 h before surgery, because of its high sugar content, but it could be used for those who injected pancreatin before meals, and oral rehydration salt, with 0.75 g potassium chloride, 1.25 g sodium bicarbonate, and 300–500 mL warm boiled water, but without sugar, could be used as a substitute.

##### 2.2.1.9 Intestinal preparation

A glycerol enema was used to induce defecation for treatment of chronic habitual constipation or no defecation for more than 2 days.

##### 2.2.1.10 Respiratory function training

To strengthen atomization, back tapping, cough and expectoration were conducted. Breathing training or balloon blowing were conducted for active and passive breathing function training.

#### 2.2.2 Intraoperative items

##### 2.2.2.1 Prevention of infection

1) First/second generation cephalosporins were used 0.5–1 h before incision. If there was excessive bleeding during the operation and the operation time was long (>3 h), an additional dose of antibacterial drugs was added during the operation. 2) During the operation, the incision was washed many times with warm saline. 3) Surgeons wore double gloves, and the surgical team frequently changed the outer gloves of the double-layer gloves.

##### 2.2.2.2 Anesthesia method

1) General anesthesia was administered by nasotracheal intubation, gauze filling of the oral cavity, and nasal application of a vasoconstrictor. 2) Compound inhalation anesthesia was administered with propofol-sufentanil-rocuronium anesthesia induction and maintenance. 3) Anesthesia and BIS anesthesia depth were monitored during surgery.

##### 2.2.2.3 Operation method

**C**ircumferential decompression of the vertebral canal and partial intralesional excision of metastatic spine tumor, using a posterolateral approach, combined with transpedicular screw implantation and internal fixation, was performed.

##### 2.2.2.4 Drainage method

Placement of drainage tube at the surgical site was limited, unless deemed necessary.

##### 2.2.2.5 Body temperature monitoring and insulation

1) Bladder temperature was monitored. 2) An electric heating pad and other surface rewarming equipment were used to maintain body temperature during operation. 3) A liquid heating device was used to warm liquids used for washing and infusion.

##### 2.2.2.6 Preemptive analgesia

Nonsteroidal anti-inflammatory drugs were administered intravenously 20 min before operation for preemptive analgesia.

##### 2.2.2.7 Local anesthesia of the incision

Subcutaneous infiltration of the long-acting local anesthetic, bupivacaine, was administered before incision and suture.

##### 2.2.2.8 Hemostasis and blood transfusion

1) Tranexamic acid (10–20 mg/kg) was injected intravenously before the operation, and was continuously pumped (1 mg/(kgh)), according to the situation during the operation. 2) During the operation, 3 g tranexamic acid was dissolved in 250 mL saline, and local tamponade used to stop bleeding after application the infiltration gauze. Intraoperative blood transfusion was only considered when hemoglobin was <70 g/L.

##### 2.2.2.9 Optimization of intraoperative fluid replacement

Goal-directed fluid therapy was recommended. During implementation of goal-directed fluid therapy, the volume reactivity index of patients was continuously and dynamically monitored, to maintain: blood pressure ≥20% of the normal value, heart rate ≥20% of the normal value, central venous pressure at 4–12 mmHg, urine volume >0.5 mL (kg h), blood lactic acid ≥2 mmol/L, central venous blood oxygen saturation <65%, and stroke volume variation ≤13%.

##### 2.2.2.10 Prevention of postoperative nausea and vomiting

1) High-risk patients were identified. 2) Multimodal analgesia was adopted to reduce the dosage of opioids and other drugs and avoid the use of inhaled anesthetics. 3) A combination of dexamethasone and tropisetron (ondansetron) was used to treat vomiting.

#### 2.2.3 Postoperative items

##### 2.2.3.1 Pain management

1) Multimodal analgesia was provided, with local anesthesia of the incision after operation, including subcutaneous infiltration of lidocaine and bupivacaine. 2) Pain killers were administered according to visual analog scale (VAS) pain score, as follows: VAS pain score <4 points, no analgesia, or oral administration of a minimum dose of non-opioid drugs; VAS pain score 4–6 points, oral or intramuscular injection, or intravenous non-opioid drugs, such as COX-2 inhibitors, was recommended; VAS pain score >7 points, patient-controlled intravenous analgesia pump, with opioid drugs including sufentanil and fentanyl.

##### 2.2.3.2 Diet management

1) Patients were given water 6 h after operation, with light oral diet, according to the patient’s tolerance, 8 h after operation, semi-liquid or solid diet 12–24 h after operation, and general diet 24–48 h after operation. 2) Patients were recommended to chew gum after operation, to promote gastrointestinal function recovery.

##### 2.2.3.3 Management of urinary catheter

If possible, the urinary catheter was removed early after operation, and routine urine bacterial culture performed at the same time.

##### 2.2.3.4 Management of drainage tube

1) Low negative pressure drainage was used for the first 6 h after operation, and the incision drainage tube was removed when drainage flow was <50 mL at 24 h after closing the negative pressure. 2) For patients with postoperative cerebrospinal fluid leakage, whose fascial layer suture tightness was uncertain, drainage tube retention time was extended, and negative pressure drainage was also prohibited.

##### 2.2.3.5 Prevention of venous thromboembolism

1) Preventive measures included basic prevention, physical prevention, and drug prevention. Basic prevention refers mainly to standardization of operation procedures and reducing operation time, and paying attention to and following information and education on the prevention of venous thrombosis and guidance on early rehabilitation exercise. Physical preventive measures included plantar vein pump, intermittent inflation compression device, and gradient pressure elastic socks. From 8 to 12 h after the operation, 0.2 mL of prophylactic low-molecular-weight heparin, such as enoxaparin, was injected subcutaneously. Low-molecular-weight heparin dose was adjusted according to patient body weight and incision drainage applied after 24 h until discharge. 3) A combination of physical prevention and drug prevention was recommended for patients with moderate and high risk of arterial thromboembolism. Physical prevention alone was applicable to patients with coagulation disorders and high risk of bleeding; a combination of physical and drug prevention was also recommended for such patients after the risk of bleeding was reduced.

##### 2.2.3.6 Management of postoperative vomiting

1) Multimodal analgesia was used to reduce the use of patient-controlled intravenous analgesia pump and opioid drugs. 2) A combination of dexamethasone and tropisetron (ondansetron) was used for preventive treatment of patients with postoperative nausea and vomiting risk scores of ≥3 points.

##### 2.2.3.7 Management of postoperative abdominal distension and constipation

1) Chewing action (such as chewing gum) can prevent postoperative abdominal distension. 2) Simotang oral liquid and lactulose oral liquid were the standard drugs used to treat postoperative abdominal distension and constipation. 3) Postoperative restriction of infusion can relieve intestinal edema caused by excessive fluid. 4) Early eating, avoiding gas-producing foods, early functional exercise, and early ambulation can prevent abdominal distension and constipation. 5) Diuretics such as furosemide and spironolactone can benefit patients with malignant ascites with high albumin gradient.

##### 2.2.3.8 Fluid management

1) Target-oriented rehydration, encouraging oral rehydration of electrolytes, and reducing intravenous rehydration. Unless the patient could not maintain urine volume or blood pressure through oral administration, intravenous infusion was not routine from the first day after surgery. 2) According to patient condition, proton pump inhibitors were individually administered and standardized.

##### 2.2.3.9 Early rehabilitation training

1) Active rehabilitation training included bed training, sitting training, standing training, and walking training. 2) Passive rehabilitation training included conventional atomization inhalation and ultrasonic vibration mechanical expectoration. 3) Patient lower limb movement was increased, to encourage calf muscle contraction. 4) Acupuncture and traditional Chinese medicine physical therapy were administered.

##### 2.2.3.10 Management and treatment of delirium

1) Symptomatic treatment was administered, based on etiology. 2) It was recommended that patients should be nursed by familiar nursing staff or family members. 3) Second-generation antipsychotic drugs, such as risperidone, olanzapine, and ziprasidone, were also used to treat delirium.

### 2.3 Surgery technique

Patients underwent circumferential decompression of the vertebral canal and partial intralesional excision of spine metastasis using a posterolateral approach. In brief, the skin was first cut along the posterior midline, under fluoroscopy, and then subcutaneous separation was performed. A percutaneous pedicle screw fixation technique was used to place the screw through the fascia. To expose the back of the spine, surgeons cut the fascia and separated the muscle through a small incision in the posterior center. The specific resection area depended on the scope of the tumor. After vertebrectomy, the vertebral body was reconstructed using Kirschner wire cement, titanium mesh, or an expandable interbody fusion cage. Finally, insertion and fixation of the intra-fascial fixation rod were completed. After operation, the muscles and deep fascia were continuously and tightly sutured with absorbable barbed thread, and the skin incision was routinely sutured intradermally.

### 2.4 Collection of data on clinical characteristics

A series of patient baseline clinical characteristics were collected, including age, sex, primary cancer, current smoking status, current alcohol consumption, comorbidities, ECOG performance score, anxiety status, and depression status. Anxiety and depression were both evaluated using HADS, which has been extensively used in patients with cancer, and its effectiveness in evaluating anxiety and depression widely tested (Annunziata et al., 2020). HADS comprises 14 items, half of which are for anxiety, and the remaining half for depression. The anxiety and depression subscales both range from 0 to 21 points, with a higher score representing higher severity of anxiety and depression. Patients with scores of 8–10 on each subscale were considered as borderline for having anxiety or depression, whereas those with scores of ≥11 were considered to have anxiety or depression. Comorbidities analyzed in this study included hypertension, diabetes, cardiovascular disease, osteoporosis, chronic lung disease, chronic liver disease, and chronic renal disease.

### 2.5 Evaluation of surgical outcomes

To comprehensively investigate the effect of ERAS on surgical outcome, a variety of clinical characteristics were collected, including surgery location, operation time, intraoperative blood loss, postoperative length of hospital stay, time to ambulation, time to regular diet, time to urinary catheter removal, time to receive radiation therapy, time to receiving systemic internal therapy (including chemotherapy, molecular targeted biological agents, immunotherapy), perioperative complications, anxiety, depression, and satisfaction with treatment. Perioperative complications included surgical site infection, spinal fluid leakage, postoperative delirium, cerebrovascular accident, cardiac arrest, and deep vein thrombosis. Anxiety and depression were both evaluated using HADS 1 week after surgery. Satisfaction with treatment was evaluated using a scoring system, with a range from 0, indicating high dissatisfaction, to 10, indicating high satisfaction, and was also assessed 1 week after surgery. Compliance with each element of the ERAS program was also evaluated, and defined as the patient meeting the requirements set out in the ERAS program.

### 2.6 Statistical analysis

Categorical data are presented as proportions, and continuous data as mean ± standard deviation (SD). Comparisons of categorical clinical characteristics were conducted using a Chi-square test, and comparisons of continuous clinical characteristics were performed using the *t*-test or Wilcoxon rank test. All statistical analyses were conducted using SAS 9.4 software. *p* values <0.05 (two-sided) were considered significant.

## 3 Results

### 3.1 Clinical characteristics of patients according to ERAS implementation

A total of 184 patients were enrolled for analysis; 98 in the non-ERAS cohort and 86 in the ERAS cohort. Baseline clinical characteristics of the two cohorts were compared and found to be comparable between the two cohorts (Table 1). Mean age was 59.94 ± 8.62 and 58.72 ± 7.69 years in the non-ERAS and ERAS cohorts, respectively (*p* = 0.316), and male patients accounted for 54.08% and 55.81% (*p* = 0.814), respectively. Lung cancer was the most common primary cancer diagnosis, with proportions of 51.02% in the non-ERAS cohort and 53.49% in the ERAS cohort (*p* = 0.961). There was also no significant difference between the two cohorts in terms of current smoker status (*p* = 0.803) or alcohol consumption (*p* = 0.909). Patients had a relatively heavy disease burden, with about half having comorbidities and almost half having an ECOG performance score ≥3. Regarding mental health, anxiety was identified in 40.82% and 44.19% of the non-ERAS and ERAS cohorts, respectively, with depression detected in 37.76% and 39.53%, respectively.

**TABLE 1:** Demographic and clinical features of patients stratified by ERAS program implementation.

Feature	Non-ERAS	ERAS	P
n	98	86	—
Age (years)	59.94 ± 8.62	58.72 ± 7.69	0.316
Sex	—	—	0.814
Male	53 (54.08%)	48 (55.81%)	—
Female	45 (45.92%)	38 (44.19%)	—
Primary cancer diagnosis	—	—	0.961
Lung	50 (51.02%)	46 (53.49%)	—
Breast	15 (15.31%)	13 (15.12%)	—
Prostate	3 (3.06%)	4 (4.65%)	—
Digestive tract	11 (11.22%)	8 (9.30%)	—
Other	19 (19.39%)	15 (17.44%)	—
Current smoker	—	—	0.803
Yes	22 (22.45%)	18 (20.93%)	—
No	76 (77.55%)	68 (79.07%)	—
Current drinker	—	—	0.909
Yes	30 (30.61%)	27 (31.40%)	—
No	68 (69.39%)	59 (68.60%)	—
Comorbidities	—	—	0.768
Yes	48 (48.98%)	44 (51.16%)	—
No	50 (51.02%)	42 (48.84%)	—
ECOG performance score	—	—	0.858
1	19 (19.39%)	15 (17.44%)	—
2	34 (34.69%)	30 (34.88%)	—
3	27 (27.55%)	28 (32.56%)	—
4	18 (18.37%)	13 (15.12%)	—
Anxiety	—	—	0.510
None	42 (42.86%)	39 (45.35%)	—
Borderline	16 (16.33%)	9 (10.47%)	—
Identified	40 (40.82%)	38 (44.19%)	—
Depression	—	—	0.546
None	44 (44.90%)	42 (48.84%)	—
Borderline	17 (17.35%)	10 (11.63%)	—
Identified	37 (37.76%)	34 (39.53%)	—

ERAS, enhanced recovery after surgery; ECOG, eastern cooperative oncology group.

### 3.2 Comparison of surgical outcomes

Surgery-related features are summarized in Table 2. Among all surgery locations, thoracic surgery was the most common, accounting for 46.94% and 51.16% in the non-ERAS and ERAS cohorts, respectively, which was not a significant difference (*p* = 0.890). Operation time was 224.99 ± 82.21 and 217.95 ± 64.91 min in the non-ERAS and ERAS cohorts, respectively (*p* = 0.524). Intraoperative blood loss was significantly higher in the non-ERAS cohort (772.36 ± 470.13 mL) than in the ERAS group (482.31 ± 319.23 mL) (*p* < 0.001).

**TABLE 2:** Surgery-related features evaluated among the two groups according to the presence of ERAS.

Feature	Non-ERAS (*n* = 98)	ERAS (*n* = 86)	*p*
Surgery location	—	—	0.890
Cervical	11 (11.22%)	7 (8.14%)	—
Thoracic	46 (46.94%)	44 (51.16%)	—
Thoracic and lumbar	14 (14.29%)	12 (13.95%)	—
Lumbar	27 (27.55%)	23 (26.74%)	—
Operation time (min)	224.99 ± 82.21	217.95 ± 64.91	0.524
Intraoperative blood loss (mL)	772.36 ± 470.13	482.31 ± 319.23	<0.001

ERAS, enhanced recovery after surgery.

Regarding postoperative outcomes, the ERAS cohort had a significantly shorter length of hospital stay than the non-ERAS cohort (5.57 ± 2.52 vs 8.27 ± 3.98 days, *p* < 0.001) (Table 3). Similar trends were also observed in terms of time to ambulation (*p* < 0.001), regular diet (*p* < 0.001), urinary catheter removal (*p* < 0.001), receiving radiation therapy (*p* < 0.001), and receiving systemic internal therapy (*p* < 0.001). The non-ERAS cohort suffered from more complications than the ERAS group (21.43% vs 9.3%, *p* = 0.24). In addition, the ERAS program could alleviate anxiety (*p* = 0.041) and improve satisfaction with treatment (*p* < 0.001), whereas it had no impact on depression (*p* = 0.415). Satisfaction score in the ERAS cohort was 8.79 ± 2.95, indicating that the majority of patients in this group were satisfied with the surgical outcomes.

**TABLE 3:** Postoperative features evaluated among the two groups according to ERAS implementation.

Feature	Non-ERAS (*n* = 98)	ERAS (*n* = 86)	*p*
Postoperative length of hospital stays (days)	8.27 ± 3.98	5.57 ± 2.52	<0.001
Time to ambulation (h)	81.34 ± 43.71	39.08 ± 17.23	<0.001
Time to regular diet (h)	35.31 ± 20.03	20.90 ± 9.09	<0.001
Time to remove urinary catheter (h)	70.15 ± 19.44	39.03 ± 15.37	<0.001
Time to receive radiation (days)	14.21 ± 5.17	7.78 ± 3.35	<0.001
Time to receive systemic internal therapy (days)	13.36 ± 4.90	8.45 ± 3.97	<0.001
Complication	—	—	0.024
Yes	21 (21.43%)	8 (9.30%)	—
No	77 (78.57%)	78 (90.70%)	—
Anxiety	—	—	0.041
None	48 (48.98%)	58 (67.44%)	—
Borderline	15 (15.31%)	8 (9.30%)	—
Identified	35 (35.71%)	20 (23.26%)	—
Depression	—	—	0.415
None	51 (52.04%)	53 (61.63%)	—
Borderline	16 (16.33%)	12 (13.95%)	—
Identified	31 (31.63%)	21 (24.42%)	—
Satisfaction score	6.06 ± 2.18	8.79 ± 2.95	<0.001

ERAS, enhanced recovery after surgery.

### 3.3 Patient compliance with ERAS interventions

Patient compliance with the ERAS program was evaluated, and the results are presented in Table 4. ERAS interventions were successfully implemented in the vast majority of patients, particularly during the preoperative and intraoperative periods; however, early removal of urinary catheter and early rehabilitation training were less than satisfactory, with only 67.44% and 72.09% compliance, respectively.

**TABLE 4:** Patient compliance with the ERAS program.

Measure	Compliance rate (%)
Pre-operation	
Comprehensive medical education	86/86 (100.00%)
Preoperative assessment	86/86 (100.00%)
Nutrition support	83/86 (96.51%)
Prevention of venous thrombosis	81/86 (94.19%)
Treatment of anemia before operation	18/18 (100.00%)
Measures to prevent bleeding	86/86 (100.00%)
Preventive pain management	80/86 (93.02%)
Diet management	86/86 (100.00%)
Intestinal preparation	86/86 (100.00%)
Respiratory function training	80/86 (93.02%)
Intra-operation	
Prevention of infection	86/86 (100.00%)
Anesthesia method	86/86 (100.00%)
Operation method	86/86 (100.00%)
Body temperature monitoring and insulation	86/86 (100.00%)
Preemptive analgesia	80/86 (93.02%)
Local anesthesia of incision	86/86 (100.00%)
Preventive hemostasis with tranexamic acid	86/86 (100.00%)
Optimization of intraoperative fluid replacement	82/86 (95.35%)
Prevention of postoperative nausea and vomiting	78/86 (90.70%)
Post-operation	
Multimodal analgesia	78/86 (90.70%)
Diet management	82/86 (95.35%)
Early removal of urinary catheter	58/86 (67.44%)
Prevention of venous thromboembolism	82/86 (95.35%)
Management of postoperative vomiting	8/8 (100.00%)
Management of postoperative abdominal distension and constipation	10/10 (100.00%)
Fluid management	80/86 (93.02%)
Early rehabilitation training	62/86 (72.09%)
Management and treatment of delirium	12/12 (100.00%)

ERAS, enhanced recovery after surgery.

## 4 Discussion

MESCC is a frequent oncological emergency and, if it is left untreated, the outcome can be catastrophic. Multidisciplinary cooperation is warranted for MESCC, with the primary therapeutic aims of alleviating pain, maintaining or restoring neurological function, and further improving patient quality of life (Wainwright et al., 2016). Surgical decompression combined with postoperative radiotherapy is considered as a standard therapeutic intervention for selected patients (Vellayappan et al., 2018), because a randomized trial demonstrated that circumferential decompressive surgery for the treatment of MESCC facilitated ambulatory status for prolonged periods of time, preserved continence, and decreased the need for steroids and opioids (Patchell et al., 2005); however, decompressive surgery for MESCC remains a challenge because of perioperative complications, intraoperative blood loss, and delayed functional recovery and postoperative radiation. Therefore, development of new medical pathways to improve the surgical outcomes of patients with MESCC is urgent.

In this study, we designed a new ERAS program, specifically tailored for patients with MESCC, based on multidisciplinary cooperation and current available medical evidence, and found that it could improve clinical metrics in terms of intraoperative blood loss; postoperative length of hospital stay; and time to ambulation, regular diet, urinary catheter removal, and receiving radiation and systemic internal therapy; as well as perioperative complications; alleviation of anxiety; and improvement of satisfaction. Hence, patients with MESCC treated using the ERAS pathway recovered heath significantly faster than those for whom the ERAS program was not implemented.

Previous studies have proved the effectiveness of ERAS in improving outcomes of spinal surgery, with promising results. For example, Ifrach et al. (Ifrach et al., 2020) investigated the effect of the ERAS pathway on elderly patients treated with spine and peripheral nerve surgery in a historically controlled study, and found that ERAS program implementation facilitated reduction in opiate use, early mobilization, and ambulation. Dagal et al. (Dagal et al., 2019) demonstrated that multidisciplinary enhanced perioperative care reduced hospital length of stay, postoperative intensive care unit, and costs for patients undergoing elective adult major spine surgery. Smith et al. (Smith et al., 2019) implemented an ERAS program for lumbar spine fusion surgery in 96 patients who received ERAS, 11 patients who were treated during the transition period, and 123 patients before the operation of ERAS. The research showed that ERAS achieved a significant reduction of opioid and rescue antiemetic use, but had a minimal effect on reducing hospital length of stay. More recently, a meta-analysis confirmed the effectiveness of ERAS in major spine surgery, finding that ERAS allowed faster recovery, lower rates of morbidity, and better long-term prognoses in patients undergoing major spine surgery (Wainwright et al., 2016). Another systematic review also showed that ERAS may decrease the rates of complication and readmissions, hospital length of stay, and use of opioids, as well as improving patient reported outcomes and functional recovery. Regarding minimally invasive spinal surgery, ERAS also showed positive effects on hospital length of stay and inpatient opioid use, according to a study in a series of 16 cases conducted by Band et al. (Band et al., 2022); however, ERAS use is limited by significant variability in reported patient populations and ERAS protocol implementation, as well as the complexity and variation of spinal procedures (Wainwright et al., 2016). Thus, it is necessary to adapt ERAS pathways for spinal procedures in specifical populations.

In the present study, we designed a novel ERAS pathway specifically for patients with MESCC. Our ERAS program impacted intraoperative blood loss, which may be attributable to the use of tranexamic acid in the ERAS pathway, as tranexamic acid is reported to decrease intraoperative blood loss during major spinal surgery (Slattery et al., 2019; Rahmani et al., 2021). In addition, the ERAS program decreased the length of hospitalization stay and complication, consistent with the results of other studies investigating the use of ERAS for spinal surgery (Dietz et al., 2019; Elsarrag et al., 2019). We also found that our ERAS program for MESCC allowed patients to recover more quickly, as it shortened the time to ambulation, regular diet, urinary catheter removal, and radiotherapy. Notably, this study is the first to report that an ERAS program had positive effects on the mental health of patients with MESCC, which may be explained by improvements in surgical outcomes and satisfaction with treatment. Compliance analysis showed that ERAS interventions were successfully operated in the vast majority of patients, and previous studies have demonstrated that higher compliance with ERAS programs contributes to better short-term outcomes, including length of stay, re-admission, and complications (Francis et al., 2018); however, early removal of urinary catheter and early rehabilitation training were less than satisfactory, and this was also consistent with the findings of other studies, as it is more difficult to obtain high levels of compliance with the postoperative elements of ERAS programs, relative to the preoperative and intraoperative elements. Therefore, more attention should be paid during the postoperative period.

### 4.1 Limitations

This study has some drawbacks. First, it was a retrospective study, hence selection bias may have affected the outcomes. Further, only 184 patients were enrolled for analysis, which is a relatively small sample size. Nevertheless, our study specifically included patients with MESCC and all patients were treated by circumferential decompressive surgery, thus the patients were a homogeneous group. Second, the new ERAS pathway was fully implemented at our institution from December 2019, and the historical characteristics of patients treated before the ERAS program was initiated served as the non-ERAS cohort. Consequently, the two cohorts were from different periods of time, and this study design may also have introduced biases. Third, our study did not investigate the long-term outcomes of patients with MESCC after receiving the ERAS program. Further studies should emphasize use of a randomized controlled trial design, assessment of long-term impacts of ERAS on surgical outcomes, and evaluation of the universal applicability of the ERAS pathway.

## 5 Conclusion

The ERAS intervention is beneficial to patients with MESCC in terms of its effects on intraoperative blood loss; postoperative length of hospital stay; time to ambulation, regular diet, urinary catheter removal, radiation, and systemic internal therapy; perioperative complications, alleviation of anxiety, and improvement of patient satisfaction. Our data suggest that the ERAS pathway can be recommended for patients with MESCC undergoing decompressive surgery; however, clinical trials to investigate the effects of ERAS are needed in the future.

## Data Availability

The raw data supporting the conclusions of this article will be made available by the authors, without undue reservation.
